# Kitchens

**Published:** 1866-04

**Authors:** 


### KITCHENS.

All persons of cultivation and refinement must instinctively shrink from cookery in the dark. Hence, it should be arranged that the sun should shine in upon this department for at least three fourths of daylight, and also that the "back-yard," as it is called, and which is usually in the rear of the kitchen, should have the advantage of abundant sunshine, so as to keep it dry and healthful.

A little sink near a kitchen door-step, inadvertently formed, has been known, although not exceeding in its dimensions a single square foot, to spread sickness through a whole household. Hence, every thing of the kind should be studiously obviated, so that there should be no spot about a farm-house which can receive and hold standing water, whether it be the pure rain from the sky, the contents of a wash-basin, the slop-bowl, or the water-pail.

